# The risk for future cerebrovascular disease in pregnant women with Moyamoya disease: a nationwide population-based study in South Korea

**DOI:** 10.1186/s12884-022-04718-8

**Published:** 2022-05-24

**Authors:** Yeonseong Jeong, Yun Ji Jung, Eunjin Noh, Sungyeon Ha, Jeongeun Hwang, Geum Joon Cho, Min-Jeong Oh, Young-Han Kim

**Affiliations:** 1 Department of Obstetrics and Gynecology, Institute of Women’s Life Medical Science, Yonsei University College of Medicine, Yonsei University Health System, Seoul, Republic of Korea; 2 Korea University Guro Hospital Smart Healthcare Center, Seoul, Republic of Korea; 3 Graduate School of Statistics, Sungkyunkwan University, Seoul, Republic of Korea; 4 Department of Biomedical Research Center, Korea University Guro Hospital, Seoul, Republic of Korea; 5 Department of Obstetrics and Gynecology, Korea University College of Medicine, Seoul, Republic of Korea

**Keywords:** Moyamoya disease, Cerebrovascular disease, Pregnancy, Nationwide large-scale study

## Abstract

**Background:**

Physiologic changes during pregnancy affect the development of postpartum cerebrovascular disease (CVD) in women with Moyamoya disease. Due to the rare prevalence of Moyamoya disease and its large regional variations, large-scale based studies on the risk of CVD after delivery have not been conducted. This study aimed to evaluate whether women with Moyamoya disease have an increased risk of CVD after delivery.

**Methods:**

Research data was collected from the National Health Insurance Claims Database of the Health Insurance Review and Assessment Service. Patients who delivered in Korea from 2007 to 2014 were enrolled in this study. We classified women as having CVD if they were diagnosed with any of the following conditions between delivery and December 31, 2016; cerebral infarction (I63.X in the International Classification of Diseases-10th Revision [ICD-10]) and/or intracranial hemorrhage (I61.X, I62.X in ICD-10) and/or subarachnoid hemorrhage (I60.X in ICD-10). Women with Moyamoya disease were identified as having I67.5 in ICD-10. We matched the study cohort by the ratio of 1:10 to analyze the risk CVD occurrence. The matching technique applied in this study was based on the variables of age and parity. To evaluate the adjusted hazard ratio (HR) for CVD in women with Moyamoya disease, we used multivariate Cox proportional hazard regression.

**Results:**

Among a total of 3,611,216 Korean women who underwent delivered, we identified 412 women with Moyamoya disease diagnosis and 1420 age- and parity-matched women without Moyamoya disease (control). Compared to the control group, women with Moyamoya disease had a significantly higher rate of Cesarean section, overt DM, and essential hypertension (all *p* < 0.0001). Among women with Moyamoya disease, 55 (13.35%) women developed CVD within the follow-up postpartum period. The presence of Moyamoya disease was associated with an increased risk of CVD after delivery (adjusted HR 37.42; 95% confidence interval (CI) 17.50-80.02 within 2.3 years) after adjusting for pregnancy-induced hypertension, gestational diabetes mellitus, pregestational diabetes, chronic hypertension.

**Conclusion:**

This population based study showed that the occurrence rate of CVD after delivery was higher in women with Moyamoya disease than in those without. Therefore, careful and long-term postpartum surveillance is required for women with Moyamoya disease.

## Background

Moyamoya disease, which is named in Japanese, is a non-inflammatory vasculopathy diagnosed through the detection of a haze on angiogram [1–3]. Moyamoya disease presents as a variety of clinical symptoms; in children, ischemic changes and seizures are the main symptoms; and in adults, cerebral hemorrhage is the main symptom [1, 4, 5]. The prevalence of Moyamoya disease is varies by region, and its prevalence rate in South Korea and Japan is higher than that in other countries [1]. In South Korea, the standardized prevalence of Moyamoya disease was 6.5 cases per 100,000 people in 2005 and the incidence was from 2.7 to 4.3 cases per 100,000 people in the same period [1, 6, 7]. Moyamoya disease is more common in women than in men [2, 3, 8, 9], at a ratio of 1.8:1 [10], and its prevalence is higher in children and in the second and third quarters of life [1]. As the disease is common among women of childbearing age [2, 9], special care is required in the management of Moyamoya disease in women before and after pregnancy.

Estrogen and progesterone are both increased during pregnancy and they promote vasodilation followed by the activation of the renin–angiotensin–aldosterone system (RAAS) [11–13]. Cerebral blood flow increases through these complex physiological changes. Pregnancy is further accompanied by hemodynamic changes, such as an increase in systemic blood flow and hypercoagulation, which increases the risk of cerebral hemorrhage in pregnant women by 5.6 times compared to that in non-pregnant women [14, 15]. Moyamoya disease patients have very fragile cerebral vessels; as a result, they are more sensitive to these physiological changes during pregnancy, and the risk of cerebrovascular disease (CVD) is higher in pregnant women with Moyamoya disease than in those without [16]. Careful management through surveillance for CVD after delivery is required in women with Moyamoya disease even if the delivery occurred without any complications.

Although studies on the risk of CVD after delivery in pregnant women with Moyamoya disease have been previously conducted [15, 17], the number of study participants has usually been very small due to the low prevalence of Moyaymoya, leaving the need for large-scale studies. Therefore, the current study aimed to evaluate the risk of CVD after delivery in pregnant women with Moyamoya disease through a national-wide cohort study.

## Methods

### Characteristics of data

This study was conducted using the Korea National Health Insurance (KNHI) claims database. In South Korea, 97% of the population are eligible to enroll in the KNHI program, excluding 3% of the population who ares under the Medical Aid Program. The KNHI claims database contains information on disease diagnosis and procedures for approximately 50 million Koreans. Therefore, almost all information about the prevalence of various diseases can be obtained from this central database [18]. According to the Act on the Protection of Personal Information Maintained by Public Agencies, The KNHI claims database do not contain individual identification information. Thus, this study was exempt from review and the requirement for informed consent was waived by the Institutional Review Board of the Korea University Medical Center (IRB No. 2020GR0160).

### Study population and outcome ascertainment

Using the KNHI claims database, we confirmed whether each subject had the diagnosis of Moyamoya disease before pregnancy based on the International Classification of Diseases-10th Revision (ICD-10) codes (I67.5).

CVD was evaluated as the primary outcome, and was defined as any of the following events: cerebral infarction, intracerebral hemorrhage, and subarachnoid hemorrhage. CVD was defined as when the following two conditions were satisfied: 1) have the corresponding diagnostic codes by ICD-10 codes between childbirth and December 31, 2016 (cerebral infarction; I63.X, intracerebral hemorrhage; I61.X, I62X, subarachnoid hemorrhage; I60.X) and 2) be hospitalized at least once. It was confirmed that CVD was not diagnosed prior to pregnancy by using the KNHI claims [19–22]. The process of developing this algorithm was confirmed by neurologists.

Based on the KNHI claims database, the following were identified as pregnancy outcomes: parity, Cesarean delivery (O82 in ICD-10 code), preeclampsia (O14.0, O14.1, and O14.9 in ICD-10 code), gestational diabetes (O24.4 in ICD-10 code), postpartum hemorrhage (O72 in ICD-10 code), placental abruption (O45 in ICD-10 code), placental previa (O44 in ICD-10 code), overt diabetes mellitus (DM, O24.0, O24.1, O24.2, O24.3, and O 24.9 in ICD-10 code) and essential hypertension (HTN, O10.0, O10.4, and O10.9 in ICD-10 code).

We also calculated the average follow-up period and person-years in both the exposed and control areas. The follow-up period started from childbirth to the date of CVD diagnosis or till the end of follow-up — i.e., December 31 2016.

### Statistical analysis

**Fig. 1:**
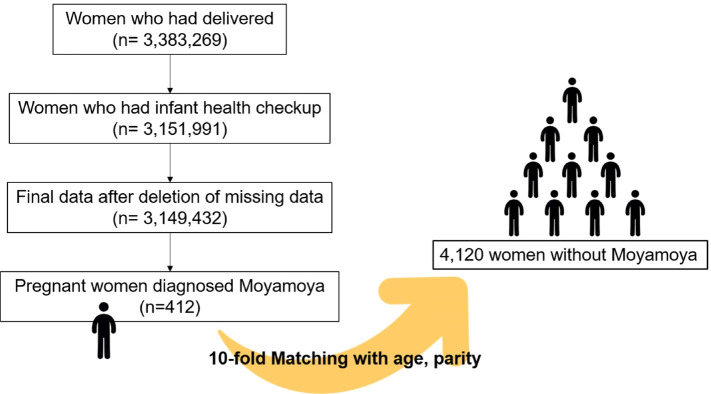
Inclusion algorithm for final study population

The study cohorts matched in a 1:10 ratio by age and parity. Differences in continuous and categorical variables were analyzed with t-test and Chi-square test, respectively. To determine the associations between Moyamoya disease and CVD risks after delivery, Cox proportional hazard models were applied to where the proportional hazard assumptions were satisfied. Otherwise, time-dependent Cox hazard models were used. A control group was formed with tenfold exact matching by age and parity for hazard analysis. In other words, 10 mothers without Moyamoya disease, whose age and parity were exactly the same with each mother with Moyamoya disease before pregnancy, were included in the control group. (Fig. 1) Schoenfeld residual test was performed to validate the proportional hazard assumption by using the “survival” package, version 3.2–13 [23–25].

A *p*-value < 0.05 was considered statistically significant. Statistical analyses were performed using SAS for Windows, version 9.4 (SAS Inc., Cary, NC, USA), and R statistics software version 4.1.1 (R Foundation for Statistical Computing, Vienna, Austria).

## Results

Out of 3,611,216 Korean women who had delivery, 412 had Moyamoya disease. After maching the study cohort by age and parity in a ratio of 1:10; 4120 controls were obtained.

Table 1. shows the sugjects’ baseline characteristics of the study population. Compared to the controls, those with Moyamoya disease had a higher rate of overt DM and essential HTN (*p* < 0.0001). In this study, Overt DM and essential HTN were defined as benign diagnosed with these conditions since1 year before delivery. Women with Moyamoya disease were more likely to undergo a cesarean section delivery (*p* < 0.0001). There was no difference in the mean maternal age between the two groups even in the subgroup analysis of women with advanced maternal age of ≥ 35 years.

**Table 1:** Comparison of Baseline Characteristics between woment with Moyamoya disease and age-parity matched control

Variables	Control group (*n* = 4,120)	Women with Moyamoya disease (*n* = 412)	*p*-value
Maternal age (y)	30.558 ± 4.130	30.558 ± 4.134	1
Advanced maternal age (> 35 y)	680 (16.5)	68 (16.5)	1
Primipara	2190 (46.84)	219 (46.84)	1
Cesarean section	1475 (35.8)	259 (62.86)	< .0001*
Essential hypertension ^a^	118 (2.86)	115 (27.91)	< .0001*
Gestational hypertension	84 (2.04)	13 (3.16)	0.1354
Overt diabetes mellitus ^a^	187 (4.54)	72 (17.48)	< .0001*
Gestational diabetes	162 (3.93)	21 (5.1)	0.2520
Postpartum hemorrhage	308 (7.48)	33 (8.01)	0.6952
Placental abruption	9 (0.22)	2 (0.49)	0.2937
Placenta previa	37 (0.9)	2 (0.49)	0.3873

Values are presented as mean ± standard deviation or N(%)

^*^P < 0.05 was considered statistically significant

^a^Diagnosis at 1 year after delivery

**Fig. 2:**
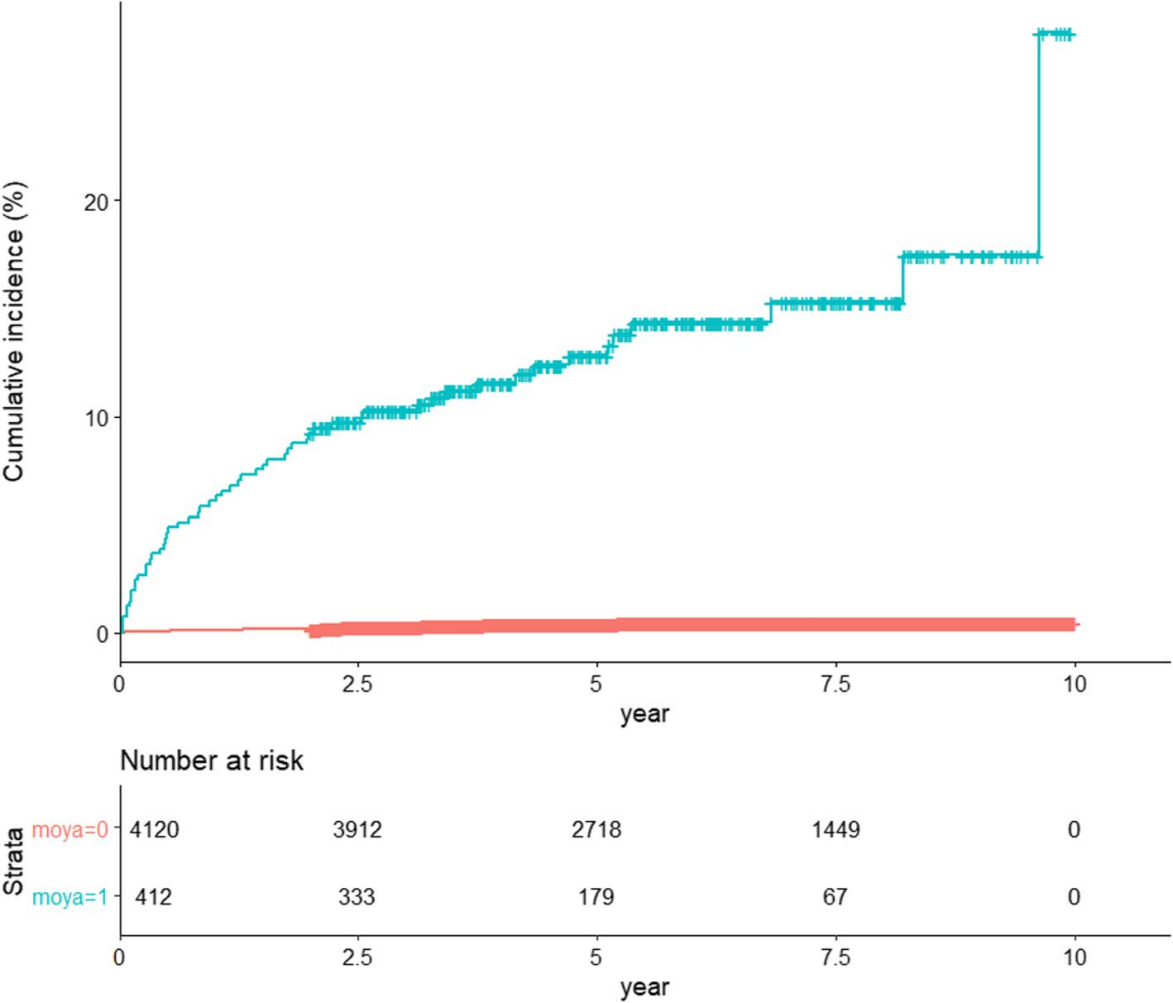
Kaplan–Meier curve depicting the effect of Moyamoya disease on the occurrence of cerebrovascular disease after delivery on time-to-event from delivery (*p* < 0.05)

The overall incidence of CVD in patients with Moyamoya disease in shown in Fig. 2. Among women with Moyamoya disease, 13.35% developed CVD after delivery, and majority of them experienced CVD within 2.3 years with an adjusted hazard ratio (HR) of 37.42 (95% confidence interval [CI] 17.50–80.02) (Tables 2 and 3). In the whole study population, the median follow-up period was 5.99 years, with CVD occurring within a median time of 1.08 year after delivery. For the control group, the median follow-up time was 6.22 years, and it took the control subjects a median of 3.63 years to develop CVD. For those in the Moyamoya disease group, the median follow-up period was 3.41 years, and it took them a median of 0.38 years from delivery to encounter CVD (Table 2).

**Table 2:** Occurrence rates of cerebrovascular disease and its subtype within the follow-up period (median follow-up time of 5.99 years)

	Control (*n* = 4,120)	Moyamoya disease (*n* = 412)	*p*-value
Cerebrovascular disease^ab^	16 (0.39)	55 (13.35)	< .0001*
Subtypes			
Cerebral infarction^§^	10 (0.24)	17 (4.13)	< .0001*
Intracerebral hemorrhage^‖^	7 (0.17)	37 (8.98)	< .0001*
Subarachnoid hemorrhage^¶^	2 (0.05)	6 (1.46)	< .0001*

Data are shown as N (%)

^*^P < 0.05 was considered statistically significant

^a^Median follow-up time: Wholestudy population: 5.99 years, Control group: 6.22 years, Moyamoya group: 3.41 years; ^b^Median follow-up times until encounter cerebrovascular disease: Study population: 1.08 years, Control: 3.63 years, Moyamoya disease: 0.38 years

^§^Median follow-up time: Whole study population: 0.84 years, Control group: 2.21 years, Moyamoya disease group: 0.61 years

^‖^Median follow-up time: Whole study population: 1.53 years, Control group: 2.16 years, Moyamoya disease group: 1.51 years

^¶^Median follow-up times: Whole Study population: 1.77 years, Control group: 1.19 years, Moyamoya disease group: 1.77 years

**Table 3:** Hazard models of developing of Cerebrovascular Disease After Delivery in Moyamoya disease

	Unadjusted HR (CI)		Adjusted HR (CI)^a^
*Cerebrovascular disease*	40.52 (23.16,70.88)	*Cerebrovascular disease within 2.3 years from delivery*	37.42 (17.50,80.02)
		*Cerebrovascular disease at 2.3 years after delivery*	20.87 (7.75,56.17)

^a^Adjusted for gestational hypertension, gestational diabetes, overt diabetes mellitus, essential hypertension

Intracerebral hemorrhage (ICH) was the leading subtype of CVD (8.98%) followed by cerebral infarction (4.13%) and subarachnoid hemorrhage (1.46%). Although the incidence rate was lower than that of patients with Moyamoya disease, CVD occurred after delivery even in women without Moyamoya disease (0.39%), with cerebral infarction having the highest incidence rate (0.24%), followed by intracerebral hemorrhage (0.17%), and subarachnoid hemorrhage (0.05%) (Table 2).

Table 3 shows the adjusted HRs of CVD in women with Moyamoya disease according to Cox proportional hazard regression analysis. In women with Moyamoya disease, the risk of CVD after delivery increased (adjusted HR 37.42; 95% confidence interval (CI) 17.50–80.02 within 2.3 years from delivery and adjusted HR 20.87; 95% confidence interval (CI) 7.75–56.17 2.3 years after delivery) after adjusting for gestational HTN, gestational diabetes, overt DM, and essential HTN. A subgroup analysis performed for each condition (intracerebral hemorrhage, cerebral infarction, and subarachnoid hemorrhage) also demonstrated similar associations. Proportional hazard assumptions were not satisfied for models of intracerebral hemorrhage; thus, time-dependent Cox regression was applied for the outcome. Intracerebral hemorrhage (adjusted HR 46.11; 95% CI 19.57–108.63) was found to have the highest increased risk of occurrence across all subgroups of CVDs, followed by cerebral infarction (adjusted HR 38.63; 95% CI 10.47–142.6 within 1.35 years and adjusted HR 4.49; 95% CI 1.02–19.75 after 1.35 years) and subarachnoid hemorrhage (adjusted HR 15.33; 95% CI 2.58–91.14) in Table 4.

**Table 4:** Risk of developing cerebrovascular disease after delivery in women with Moyamoya disease according to its subtypes

	Unadjusted HR (CI)		Adjusted HR (CI)*
*Cerebral infarction*	18.67 (8.52,40.94)	*Cerebral infarction within 1.35 years from delivery*	38.63 (10.47,142.6)
		*Cerebral infarction at 1.35 years after delivery*	4.49 (1.02,19.75)
*Intracerebral hemorrhage*	63.87 (28.35,143.9)		46.11 (19.57,108.63)
*Subarachnoid hemorrhage*	31.23 (6.30,154.8)		15.33 (2.58,91.14)

^*^Adjusted for gestational hypertension, gestational diabetes, overt diabetes mellitus, essential hypertension

## Discussion

The present nationwide study has confirmed that the risk of CVD increases after delivery in women with Moyamoya disease. Although there have been several studies on CVD during pregnancy and after childbirth in pregnant women with Moyamoya disease, no large-scale studies have been conducted yet, due to the low prevalence of the disease itself [15, 17, 26].

The diagnosis of Moyamoya disease has been increasing in recent years, which can be presumed to be due to an increase in accessibility to imaging study. According to previous studies, cerebral hemorrhage in pregnant women with Moyamoya disease occurs mainly in the second trimester of pregnancy, and cerebral ischemia occurs mostly after delivery [10]. Although the cause of CVD after delivery in women with Moyamoya disease has not yet been fully investigated, a possible mechanism can be considered when an increased cardiac output is maintained 24–48 h after delivery and returns to normal within 10 days postpartum. It is hypothesized that the increased coagulability and the decreased in cerebral blood flow following the lower cardiac output cause ischemic cerebral disease after delivery in women with Moyamoya [2, 13, 14]. Moreover, blood flow, which increases rapidly by the second trimester of pregnancy, decreases and increases again by the 24–26 weeks of gestation. This is predicted to be the cause of the increase risk for cerebral hemorrhage. Another hypothesis of causality is the strength of the blood vessels. The women undergone delivery have rapid hormonal and hemodynamic changes that affect the coagulation system which makes blood vessels fragile. This phenomenon indicates that the delivery is a risk factor of CVD itself. Furthermore, our study showed that the women with MMD have an increased risk of future CVD following delivery even if CVD would not occur immediately after delivery. However, as mentioned previously, the causality of the occurrence of the CVD after delivery in women with Moyamoya disease has not yet been fully evaluated; hence, further studies are required [27, 28].

The present study included only women who were diagnosed with Moyamoya disease before pregnancy. Diagnosis of Moyamoya disease during pregnancy resulted in worse prognosis than those diagnosed before pregnancy [11, 29, 30]. This is thought to be due to the stricter blood pressure control and the administration of appropriate treatment for women diagnosed with Moyamoya disease before pregnancy [11]. On the other hand, the diagnosis of Moyamoya disease during pregnancy is usually due to a cerebrovascular event, which leads to a poorer outcome. In a previous nationwide survey conducted in Japan, among 64 pregnant women with Moyamoya, five were newly diagnosed during pregnancy due to cerebral events [11].

In this study, pregnant women with Moyamoya disease showed significantly higher rates of Cesarean section compared to generally healthy women, because such approach is preferred as a delivery method for women with Moyamoya disease [10, 11]. Hemodynamic changes that can occur during labor include an elevation in the blood pressure due to the Valsalva maneuver and a decrease in cerebrovascular blood flow due to hypercapnia caused by hyperventilation [2, 10]. These physiological alterations are likely to cause problems in women with Moyamoya disease, who have weaker blood vessels than generally healthy women. Another reason for the high Cesarean section rate can be explained by the significantly higher rate of associated diseases, such as pregnancy-induced HTN and gestational diabetes in women with Moyamoya disease. However, a recent study on vaginal delivery without complications in pregnant women with Moyamoya disease reported that maintaining stable vital signs was more relevant to the prognosis of pregnant women with Moyamoya disease than the mode of delivery [5, 30, 31]. However, studies comparing the results of cesarean section with those of vaginal delivery have not been conducted yet. Several recent studies have investigated whether the stroke risk varies with the delivery method [11, 32]. A study concluded that there were no complications with vaginal delivery in pregnant women with Moyamoya disease under epidural anesthesia [32–34]. Studies have shown that if the women showed normal cerebral circulation on single-photon emission computed tomography at 1 year before pregnancy, vaginal delivery under epidural anesthesia is worth trying. The common conclusion across all these studies is that a safe vaginal delivery attempts are possible only when the cerebral blood flow is stable [3, 32].

CVD is a major contributor to dementia; hence, we consider Moyamoya disease as a risk for future dementia. However, the average age of dementia patients worldwide is over 65 years, and the subjects in the current study, who are in the reproductive era of their lives; thus, they are relatively too young to develop dementia even after 10 years.

Several limitations should be considered when interpreting the findings of the present study. First, this study was based on the insurance claim data from the KNHI Claims Database, which was designed for cost claim issues, not research. Therefore, information on the exact cause of CVD was not available. Furthermore, this study could not consider specific situations about dealing with Moyamoya disease patients. Asymptomatic infarct or microbleeds detected only on the imaging study can affect the consequences of the Moyamoya disease patients but could not be excluded in this study because of the limitation of the large scale based data and the CVD coded only ICD-10 system. If transient ischemic attack, which is a common phenomenon of Moyamoya disease, had been taken into account when defining cerebral infarction, more accurate results may have been derived. Second, due to the nature of retrospective observation studies, in the case of CVD in pregnant women without Moyamoya disease, there was no review of whether Moyamoya disease was present but underdiagnosed. Third, this study did not take note of whether the woman with Moyamoya disease received appropriate treatment at the time of delivery in cases where CVD occurred in the acute phase after delivery. Moreover, this study did not consider the traditional risk factors related to CVD including smoking, excessive alcohol drinking, marital status, physical inactivity, socioeconomic status, and comorbidities, such as psychiatric disorders, owing to the limitation of being a large-scale based studies. Finally, this study showed the occurrence of CVD in women after delivery within at least 7 years. The present study did not consider past bypass surgery, pre-pregnancy severity of Moyamoya disease, or how adequately the cases were managed before pregnancy. Further study is needed about this issue. If future studies will include these problems in their analysis, more accurate data about the occurrence of CVD after delivery in women with Moyamoya disease will be obtained, allowing these women to decide on their child-bearing plans.

Despite these limitations, this study was still significant in that it was a large-scale study on CVD in pregnant women with Moyamoya disease, and demonstrating consistency with previous studies. Although it is well known that pregnant women with Moyamoya disease have a higher risk of developing CVD compared to generally healthy subjects, no research has been conducted on the postpartum follow-up period; and further studies are required on this matter.

## Conclusion

In this study, most of the CVD events occurred within a few years of deliver in women with Moyamoya disease. However, even if a pregnant woman with Moyamoya disease delivered normally, CVD may occur after a fairly long time, suggesting that a long-term management for this patient group is necessary.

## Data Availability

The datasets used in the present study are available from the corresponding author (yhkim522@yuhs.ac, md_cho@hanmail.net) only upon reasonable request.

## Abbreviations

KNHI: Korea National Health Insurance
CVD: Cerebrovacular disease
HR: Hazard ratio
ICD-10: International Classification of Diseases—10th Revision
DM: Diabetes mellitus
HTN: Hypertension
CI: Confidence interval
